# Supplementary material for: Lamin A/C Is Dispensable to Mechanical Repression of Adipogenesis
Source: Int J Mol Sci. 2021 Jun 19;22(12):6580. doi: 10.3390/ijms22126580 (PMC8234021; doi:10.3390/ijms22126580)
Supplement: Supplementary file 1 [file ijms-22-06580-s001.zip › ijms-1245014-supplementary.pdf]

Supplementary Information

Lamin A/C is dispensable to mechanical repression of adipogenesis

Matthew Goelzer[1], Amel Dudakovic[2], Melis Olcum[3, 4], Buer Sen[3], Engin Ozcivici[4], Janet Rubin[3], Andre van Wijnen[2], Gunes Uzer[1 †]

[1]Boise State University, [2]Mayo Clinic, [3]University of North Carolina Chapel Hill, [4]Izmir Institute of Technology

† Corresponding Author

Funding support: NIH AG059923 & P20GM109095, NSF 1929188 & 2025505 (GU), R01AR049069 (AJvW), AR075803 (JR), Career Development Award in Orthopedics Research (AD), The Scientific and Technological Research Council of Turkey 2214-A (MO).

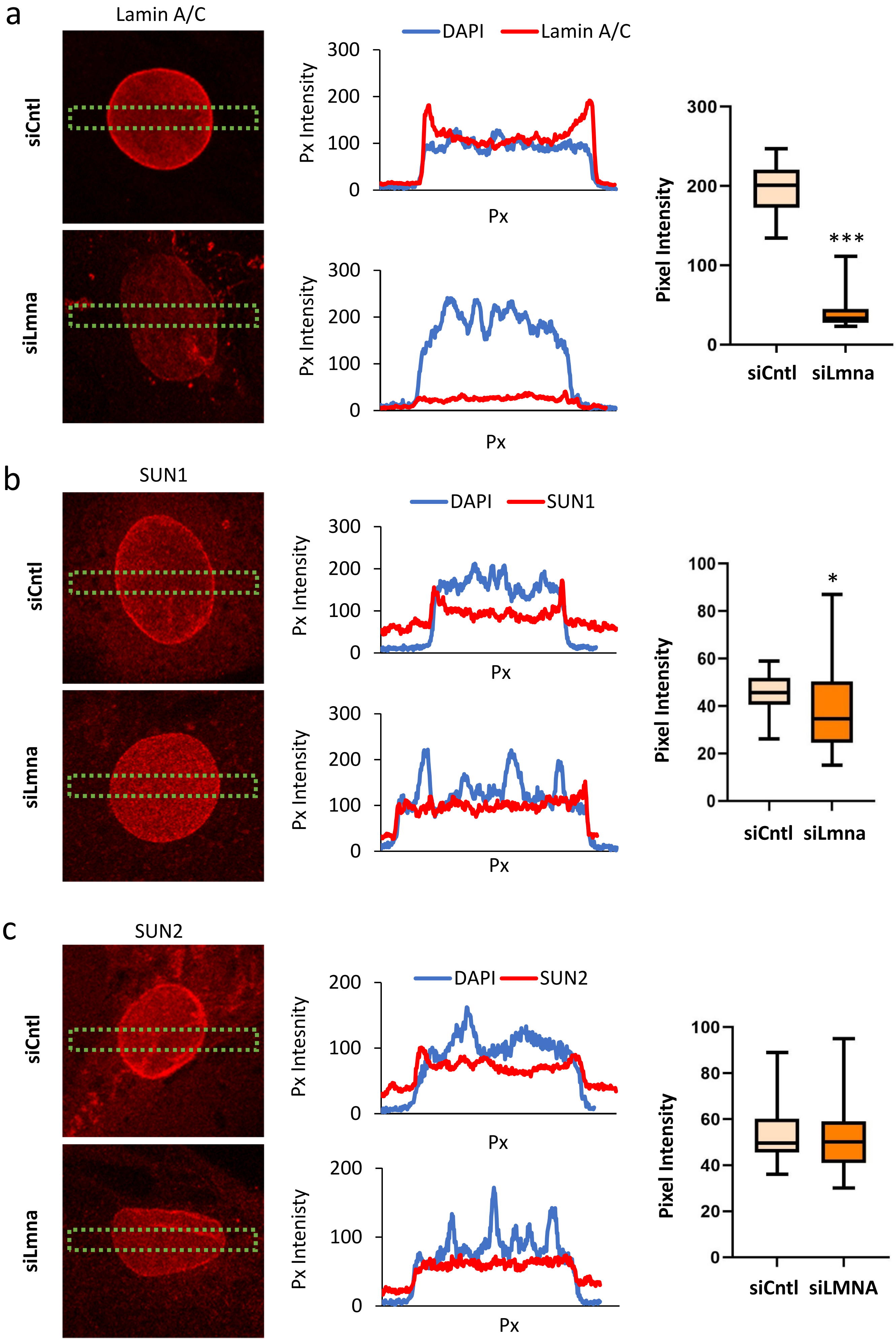

a
Lamin A/C
siCntl
siLmna
DAPI
Lamin A/C
Px Intensity
Px
Pixel Intensity
siCntl
siLmna
***
b
SUN1
siCntl
siLmna
DAPI
SUN1
Px Intensity
Px
Pixel Intensity
*
siCntl
siLmna
c
SUN2
siCntl
siLmna
DAPI
SUN2
Px Intesnity
Px
Px Intenisty
Pixel Intensity
siCntl
siLMNA

Fig. S1 Quantification of confocal images for SUN1, SUN2 and lamin A/C in siCntl and siLmna groups. **a** Intensity profile of lamin A/C staining along a rectangular region of interest on the nucleus. The middle plot shows the representative intensity distribution of lamin A/C over the nucleus (blue, Hoechst 33342). Lamin A/C intensity peaked at the nuclear rim in siCntl cells while no peaks were observed in siLmna cells. Comparison of peak intensity values at the nuclear envelope show 80% ($p<0.001$, n=25/grp) decrease with siLmna treatment. **b** Intensity profile of SUN1 staining along a rectangular region of interest on the nucleus. The middle plot shows the representative intensity distribution of SUN1 (red) over the nucleus (blue, Hoechst 33342). Comparison of peak intensity values at the nuclear envelope show 15% ($p<0.05$, n=19/grp) decrease with siLmna treatment. **c** Intensity profile of SUN2 staining along a rectangular region of interest on the nucleus. The middle plot shows the representative intensity distribution of SUN2 (red) over the nucleus (blue, Hoechst 33342). No difference between siCntl and siLmna was detected. Images were quantified using ImageJ. Results are presented as mean ± SD. Group comparisons were made via non-parametric Mann Whitney U-test. * $p<0.05$, ** $p<0.01$, *** $p<0.001$.

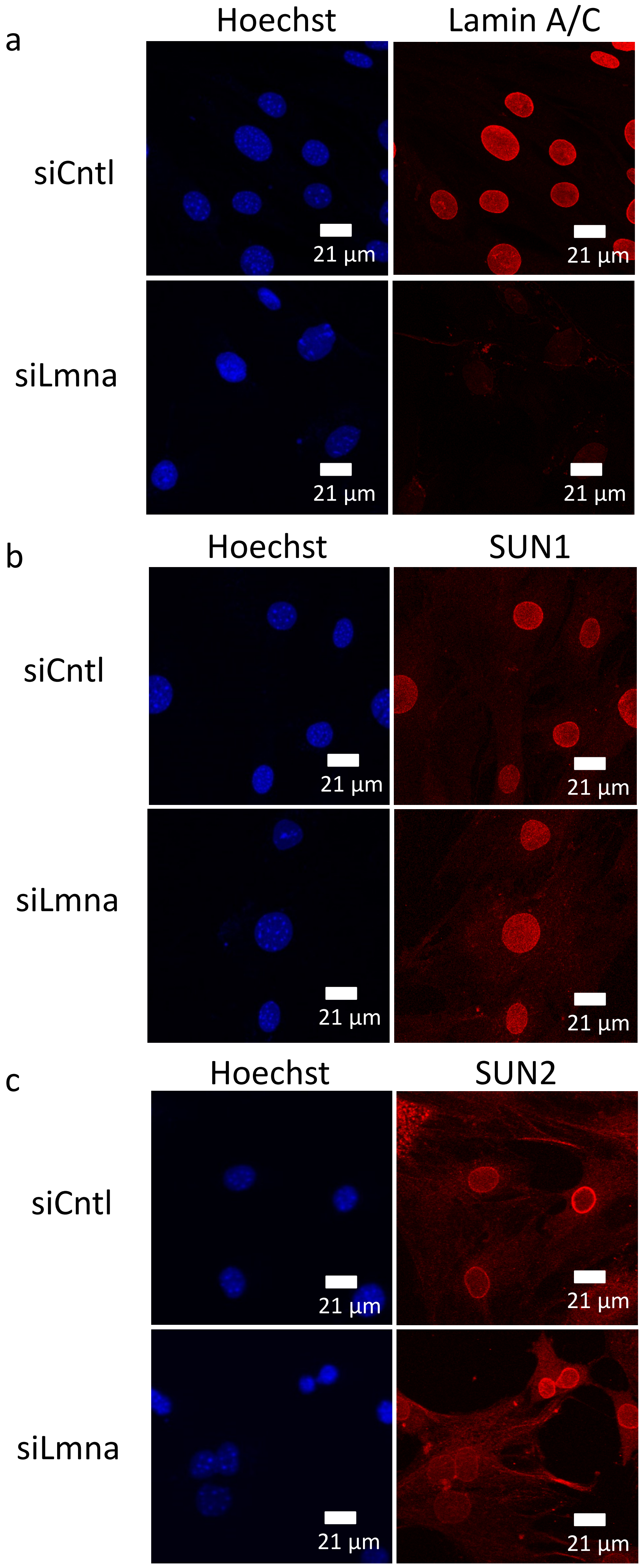

Fig. S2 Representative photos of nuclei (blue, Hoechst 33342) for Figure 2a. **a** lamin A/C (Red, 594nm) **b** SUN1 (Red, 594nm) **c** SUN2 (Red, 594nm). Scale bars: 21µm

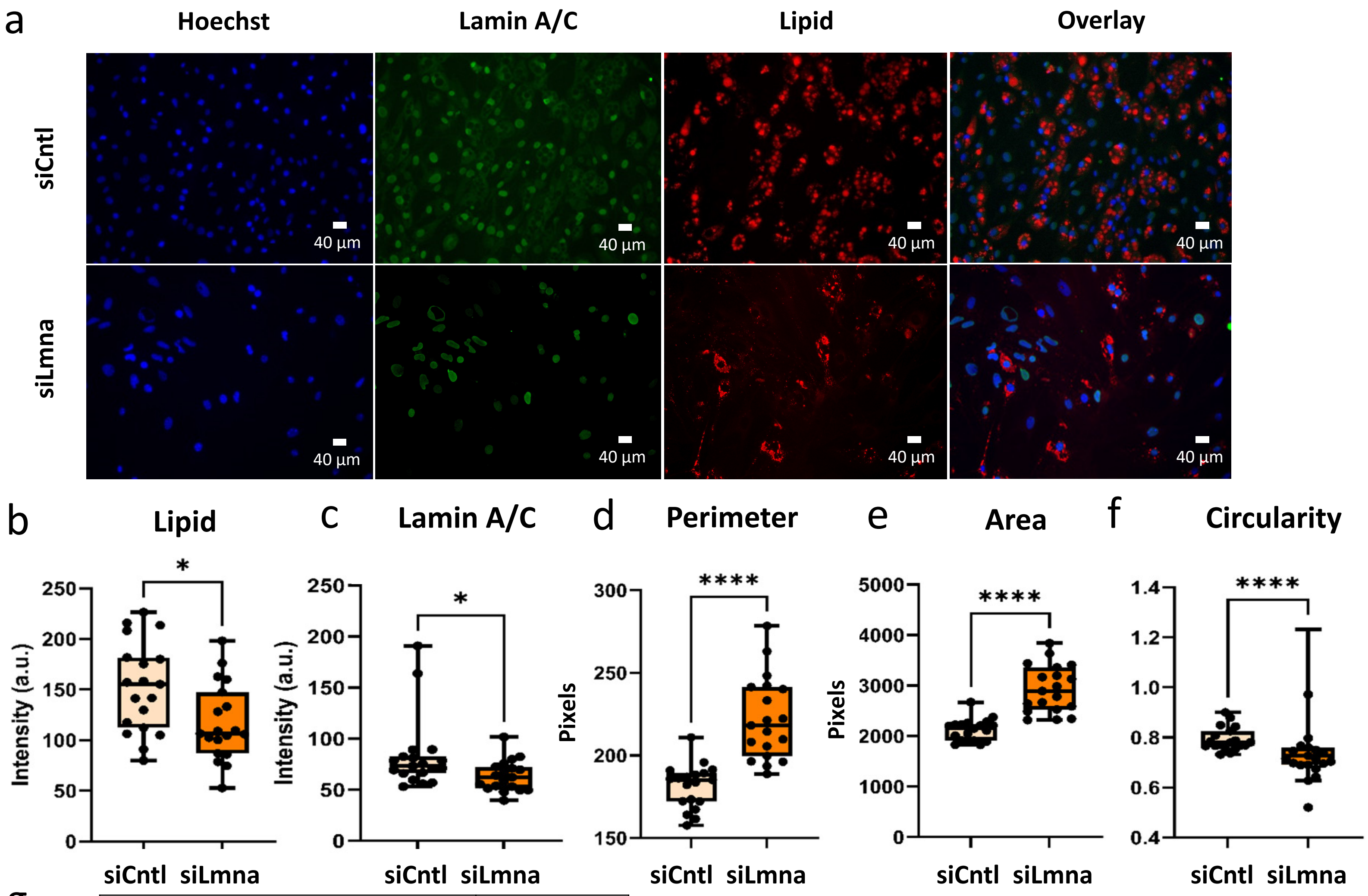

g

Analysis of Variance	P value	
Lamin A/C	P=0.9036	
Area	P=0.0927	
Perimeter	P=0.0291	*
Circularity	P=0.4937	
Lamin A/C : Area	P=0.0270	*
Lamin A/C : Perimeter	P=0.0181	*
Lamin A/C : Circularity	P=0.3389	
Area : Perimeter	P=0.7455	
Area : Circularity	P=0.0873	
Perimeter : Circularity	P=0.0321	*
Lamin A/C : Area : Perimeter	P=0.2590	
Lamin A/C : Area : Circularity	P=0.0265	*
Lamin A/C : Perimeter : Circularity	P=0.0191	*
Area : Perimeter : Circularity	P=0.8447	

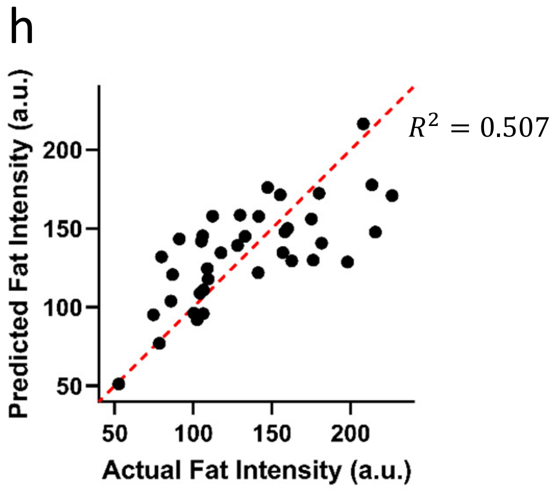

Fig. S3 Quantification of lamin A/C, lipid levels and, cell structural parameters during adipogenesis in siCntl and siLmna groups. a Representative photos of Nuclei (blue, Hoechst 33342), lamin A/C (green, 488nm), and Lipid (Red, 610nm) staining for siCntl and siLmna cells. Scale bars: 40µm. **b** Measurement of average pixel intensity of lipid droplets per cell. siLmna treated cells had 23% less intensity then siCntl treated cells (n=19 , p<0.05). **c** Average intensity of Lamin A/C. siLmna treated cells had 24% less intensity then siCntl treated cells (n=19, p<0.05). **d** Average perimeter of nuclei measured in pixels per image. siLmna cells had 32% greater perimeter then siCntl cells (n=19, p<0.0001). **e** Average nuclei per area per image as measured in pixels. siLmna cells had 38% greater area than siCntl cells (n=19, p<0.0001). Average circularity per image. siLmna cells had a 11% decrease in circularity compared to siCntl cells (n=19, p<0.0001). **g** Anova analysis of parameters for multiple linear regression model. Perimeter, Lamin A/C: Area, Lamin A/C: Perimeter, Lamin A/C: Area: Circularity, and Lamin A/C Circularity : Perimeter comparisons were found to be significant (p<0.05). **h** Multiple linear regression model with main affects, two-way, and three-way interactions predicting average lipid droplet intensity per cell ($R^2 = 0.507$). * p<0.05, ** p<0.01, *** p<0.001.

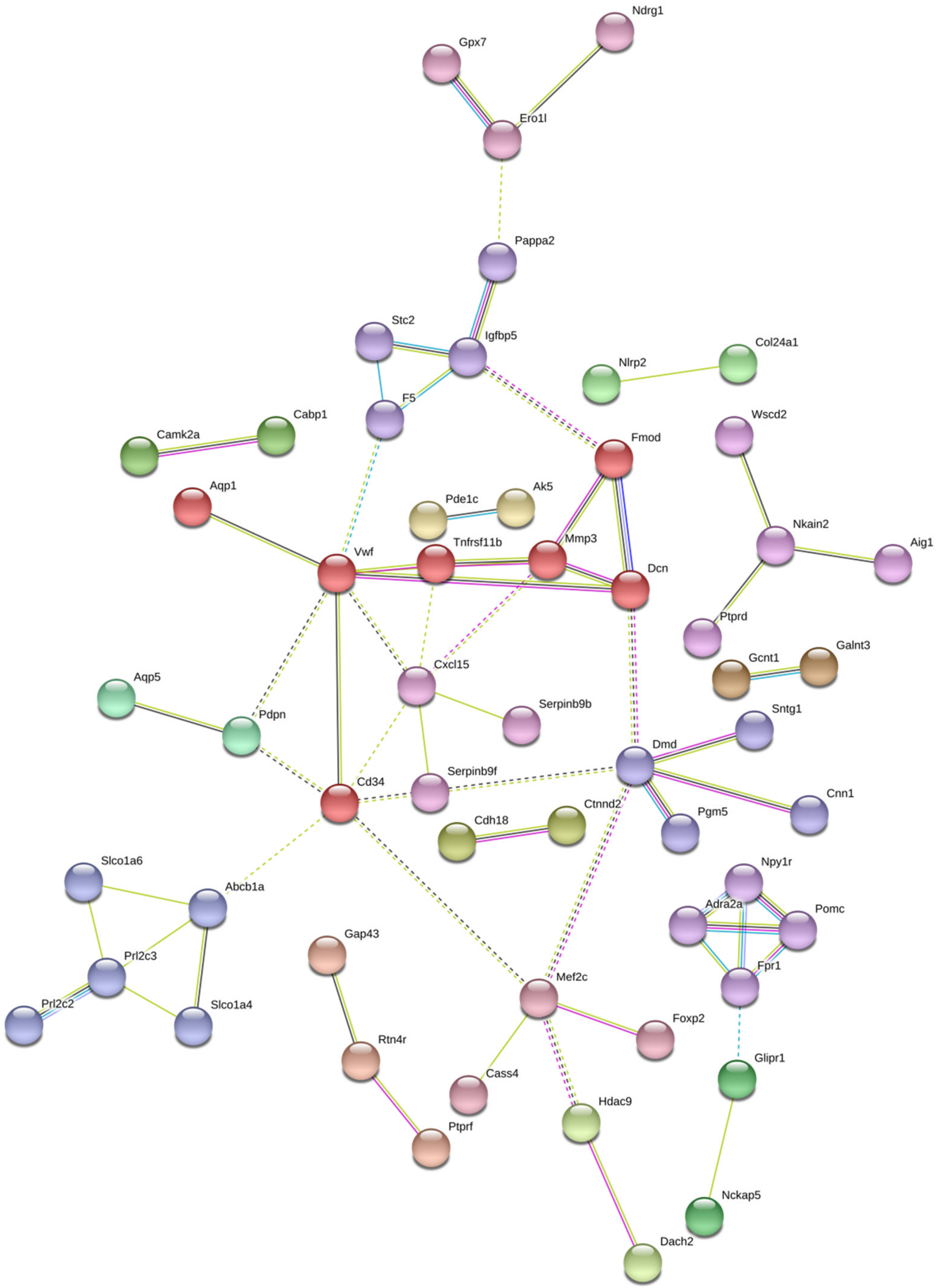

Fig. S4 Full size, annotated gene cluster for Fig.6b.

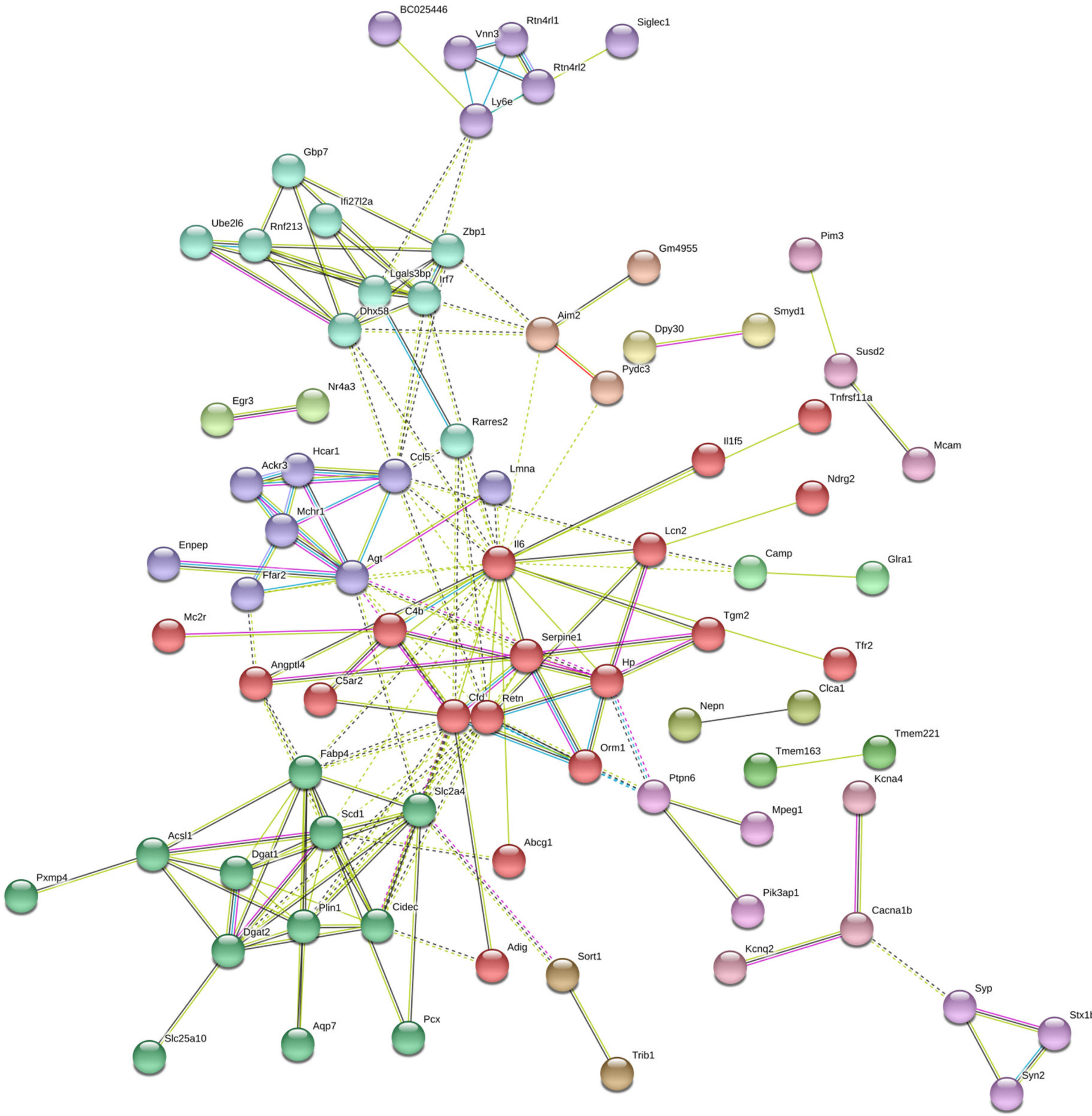

Fig. S5 Full size, annotated gene cluster for Fig.6d

Fig. S4

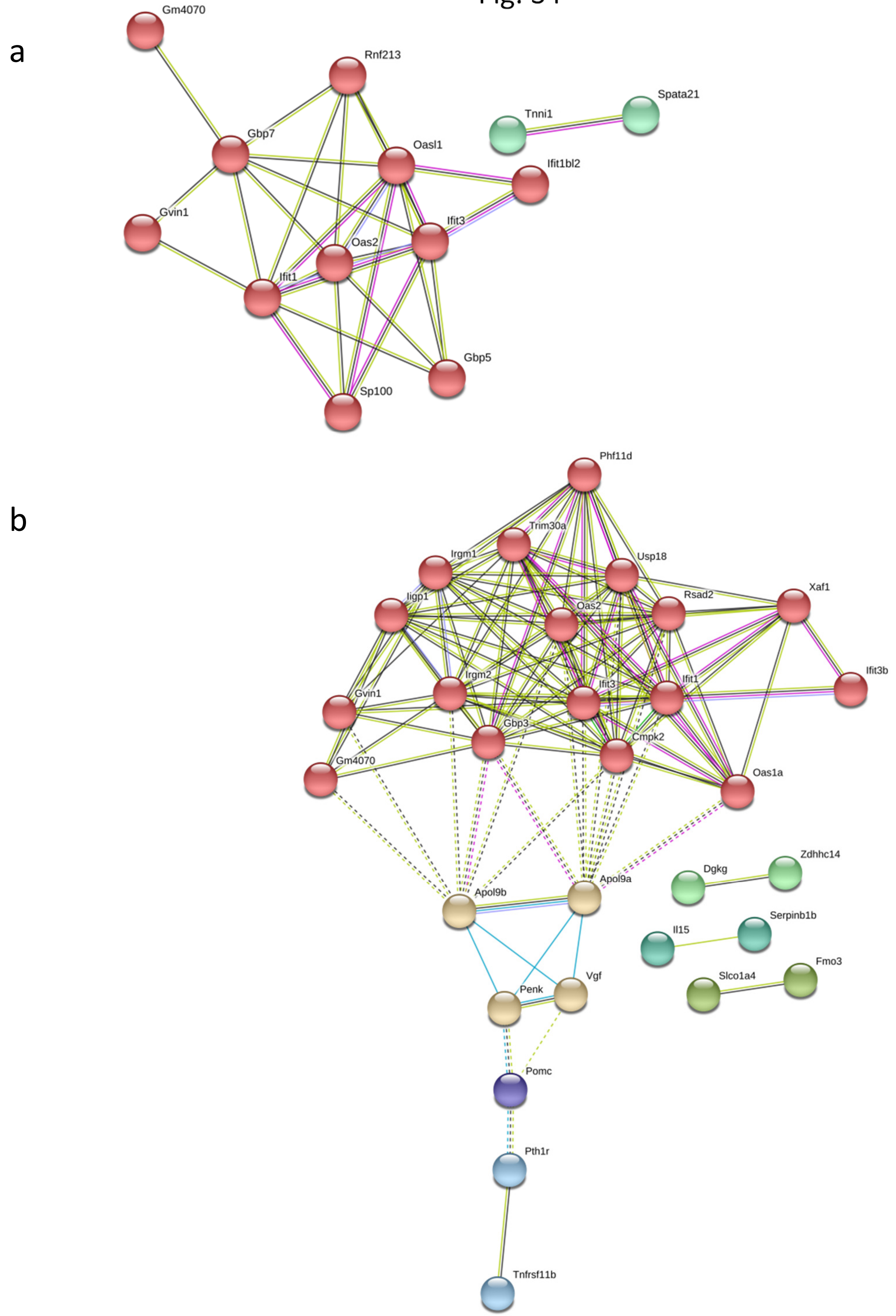

Fig. S6 Full size, annotated gene cluster for **a** Fig.7c and **b** Fig.7d

a

Tissue repair
Aqp1<br>Vwf<br>Tnfrsf11b<br>Mmp3<br>Dcn<br>Cd34<br>Fmod

ECM remodeling & cell surface transporters
slco1a6<br>Prl2c2<br>Prl2c3<br>Abcb1a<br>slco1a4

b

Cell adhesion & cytoskeletal organization	
Bc025446<br>Vnn3<br>Ly6e	Rtn4rl1<br>Rtn4rl2<br>Siglec1

Interferon signaling & regulation of gene expression	
Ube2l6<br>Rnf213<br>Gbp7<br>Ifi27l2a	Zbp1<br>Irf7<br>Lgals3bp<br>dhx58

G protein coupled receptor signaling	
Hcar1<br>Ackr3<br>Ccl5<br>Mchr1	Enpep<br>Ffar2<br>Agt<br>Lmna

Lipid metabolism & inflammatory signaling	
Tnfrsf11a<br>Il1f5<br>Ndrg2<br>Lcn2<br>Tfr2<br>Tgm2<br>Hp<br>Orm1	Serpine1<br>Retn<br>Abcg1<br>Cfd<br>Angptl4<br>C4b<br>C5ar2<br>Mc2r

Adipogenesis	
Fabp4<br>Slc2a4<br>Scd1<br>Dgat1<br>Plin1<br>cidec	Pcx<br>aqp7<br>Slc25a10<br>Acsl1<br>Pxmp4

Fig. S7 Gene lists for **a** Fig.6b and **b** Fig.6c

Sun-1

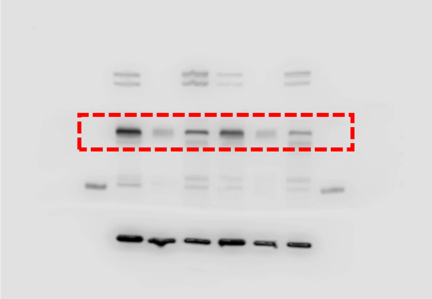

LDHA

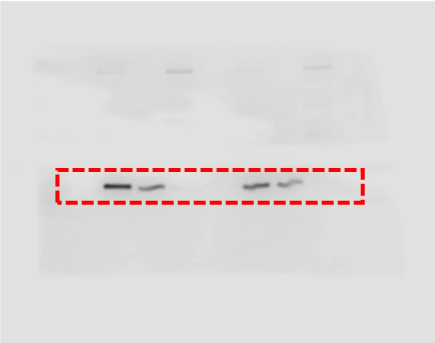

PARP

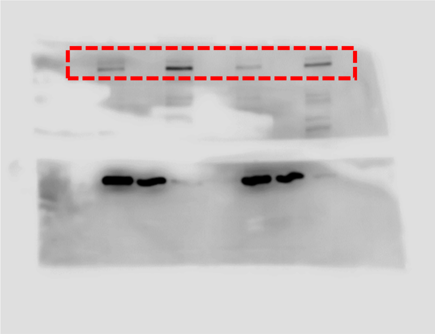

Lamin A/C

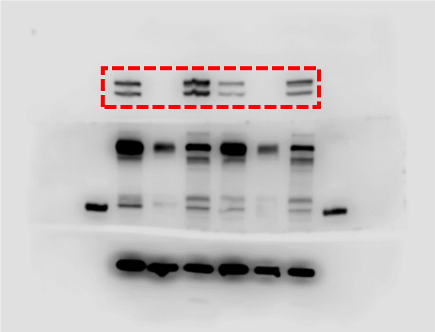

Sun-2

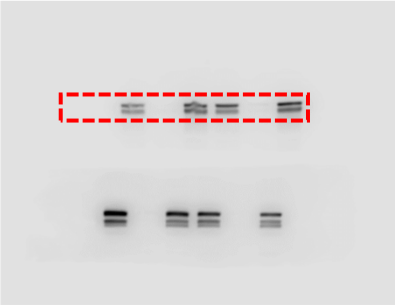

FAK

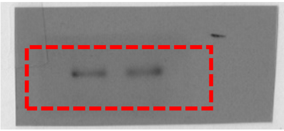

Akt

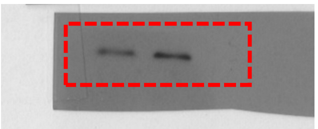

Vinculin

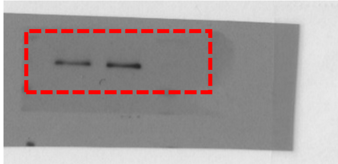

Figure S8. Unprocessed blots used in Figure 2 as obtained by LiCor C-DiGit blot scanner.

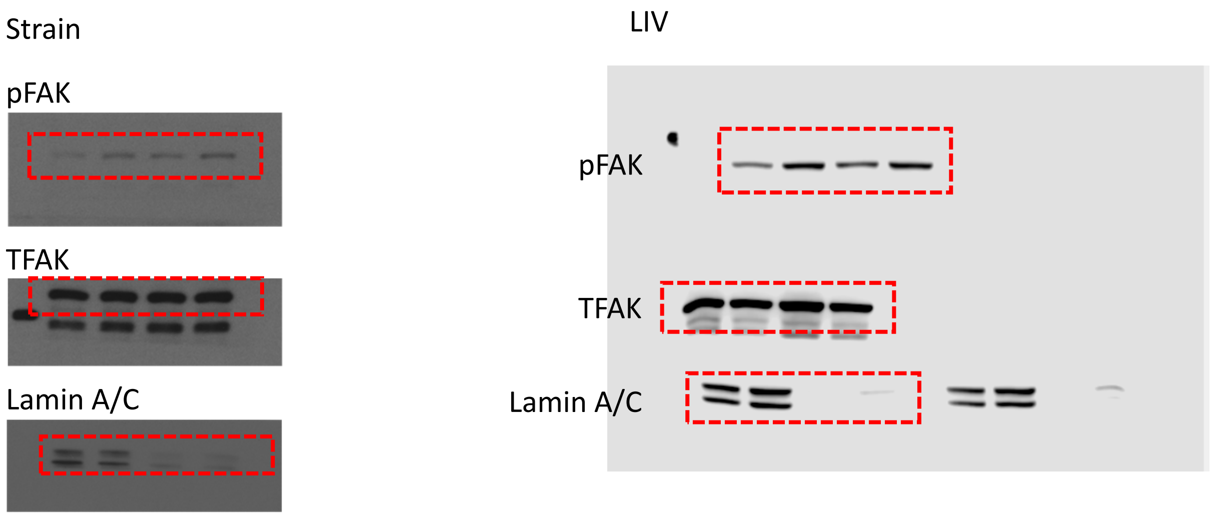

Figure S9. Unprocessed blots used in Figure 3 as obtained by LiCor C-DiGit blot scanner.

β-tubulin

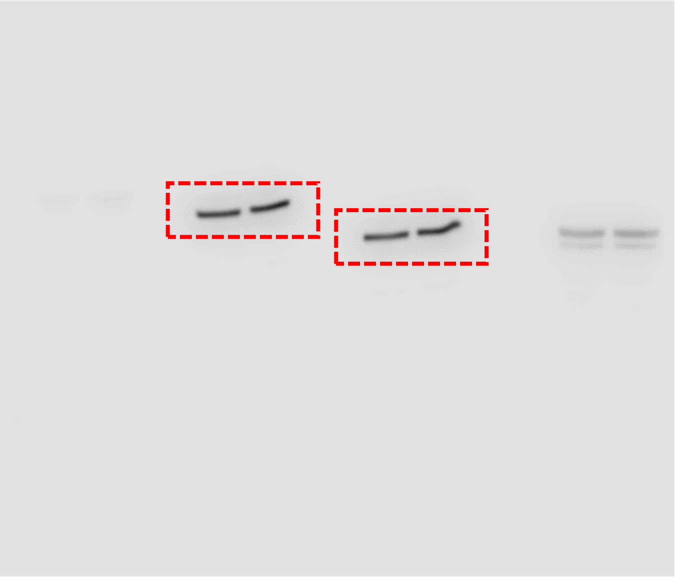

Lamin A/C

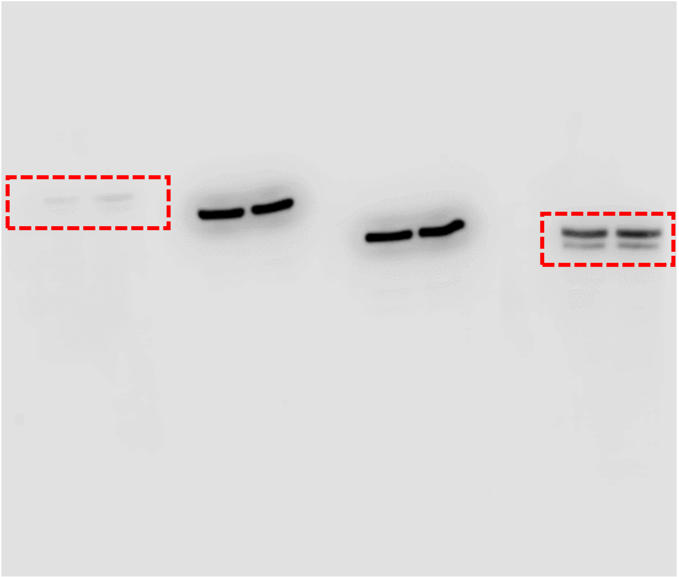

APN

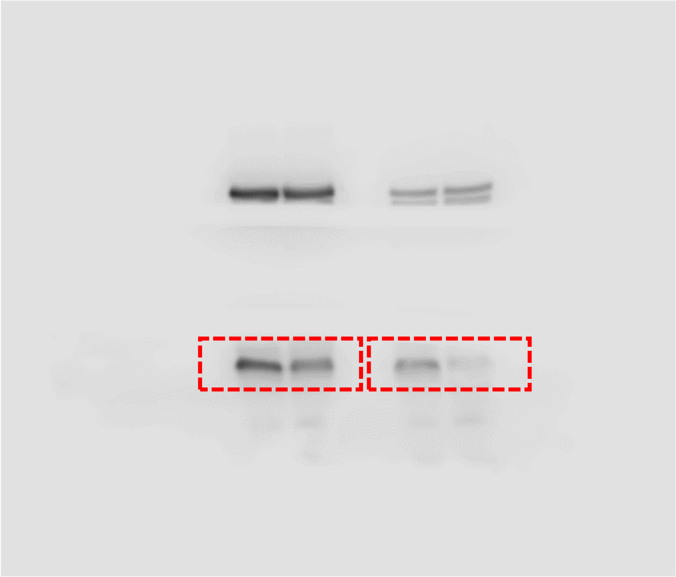

Figure S10. Unprocessed blots used in Figure 4 as obtained by LiCor C-DiGit blot scanner.

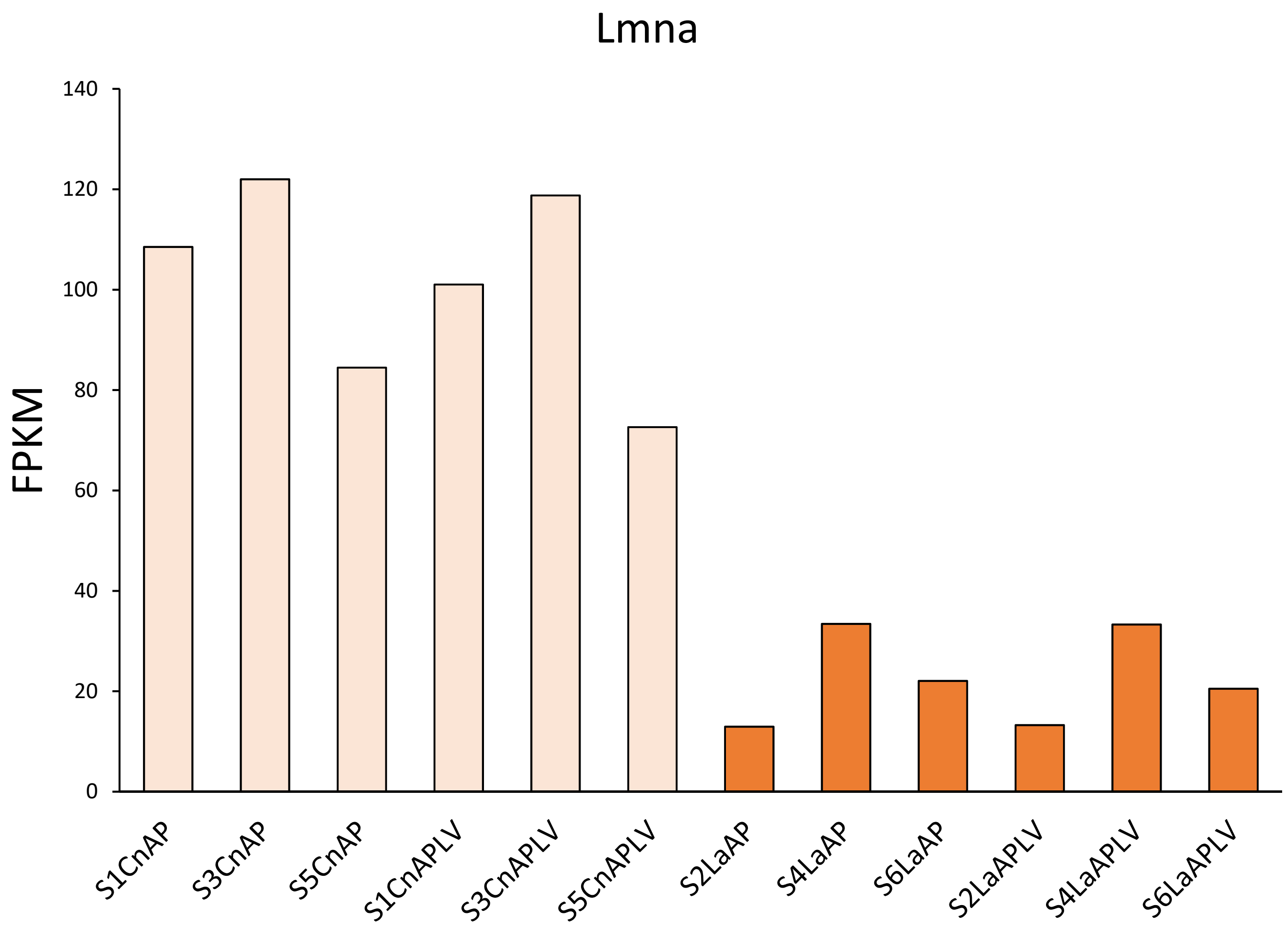

Fig. S11. Lmna FPKM levels for RNA-seq samples.

Table S1: Table of biological and cellular pathways with an FDR < 0.05 detected in STRING analysis from Fig. 6b.

#term ID	term description	strength	false discovery rate
GO:0005576	extracellular region	0.45	5.96E-06
GO:0005886	plasma membrane	0.24	0.0097
GO:0005615	extracellular space	0.41	0.0169
GO:0030054	cell junction	0.43	0.0169
GO:0031527	filopodium membrane	1.46	0.0169
GO:0042383	sarcolemma	0.82	0.0169
GO:0098590	plasma membrane region	0.4	0.0208
GO:0005887	integral component of plasma membrane	0.37	0.0462
GO:0016324	apical plasma membrane	0.59	0.0462
GO:0032501	multicellular organismal process	0.23	0.0084
GO:0042060	wound healing	0.77	0.0084
GO:0003008	system process	0.39	0.0278
GO:0022603	regulation of anatomical structure morphogenesis	0.46	0.0278
GO:0032502	developmental process	0.21	0.0278
GO:0032879	regulation of localization	0.31	0.0278
GO:0048856	anatomical structure development	0.23	0.0278
GO:0050878	regulation of body fluid levels	0.71	0.0278
GO:0050896	response to stimulus	0.18	0.0278
GO:0007275	multicellular organism development	0.22	0.0308
GO:0048731	system development	0.24	0.0308
GO:0007586	digestion	0.99	0.0419

Table S2: Table of top 72 biological and cellular pathways with an FDR < 0.05 detected in STRING analysis from Fig. 6d.

#term ID	term description	strength	false discovery rate
GO:0010646	regulation of cell communication	0.4	2.14E-06
GO:0023051	regulation of signaling	0.39	2.14E-06
GO:0048583	regulation of response to stimulus	0.34	4.37E-05
GO:0065007	biological regulation	0.18	4.37E-05
GO:0050789	regulation of biological process	0.18	5.29E-05
GO:0031349	positive regulation of defense response	0.86	7.66E-05
GO:0050794	regulation of cellular process	0.19	7.66E-05
GO:0006952	defense response	0.54	8.60E-05
GO:0009966	regulation of signal transduction	0.36	8.60E-05
GO:0071345	cellular response to cytokine stimulus	0.64	8.60E-05
GO:0006955	immune response	0.55	0.00015
GO:0007154	cell communication	0.3	0.00015
GO:0009605	response to external stimulus	0.41	0.00015
GO:0010648	negative regulation of cell communication	0.49	0.00015
GO:0023052	signaling	0.3	0.00015
GO:0023057	negative regulation of signaling	0.49	0.00015
GO:0045444	fat cell differentiation	1.05	0.00015
GO:0048518	positive regulation of biological process	0.25	0.00015
GO:0050896	response to stimulus	0.22	0.00015
GO:0051707	response to other organism	0.53	0.00015
GO:0098542	defense response to other organism	0.6	0.00015
GO:0032103	positive regulation of response to external stimulus	0.72	0.00021
GO:0048584	positive regulation of response to stimulus	0.4	0.00023
GO:0009968	negative regulation of signal transduction	0.49	0.00027
GO:0048519	negative regulation of biological process	0.26	0.00039
GO:0050873	brown fat cell differentiation	1.38	0.00039
GO:0007166	cell surface receptor signaling pathway	0.42	0.00042
GO:0019221	cytokine-mediated signaling pathway	0.76	0.00048
GO:0032879	regulation of localization	0.34	0.00048
GO:0048585	negative regulation of response to stimulus	0.43	0.00048
GO:0006954	inflammatory response	0.67	0.00052
GO:0002684	positive regulation of immune system process	0.55	0.00056
GO:0019216	regulation of lipid metabolic process	0.73	0.00066
GO:0070887	cellular response to chemical stimulus	0.36	0.00066
GO:0045834	positive regulation of lipid metabolic process	0.91	0.00068
GO:0071356	cellular response to tumor necrosis factor	0.91	0.0007

GO:0048660	regulation of smooth muscle cell proliferation	0.91	0.00071
GO:0050729	positive regulation of inflammatory response	0.99	0.00077
GO:0051049	regulation of transport	0.39	0.00085
GO:0032101	regulation of response to external stimulus	0.53	0.00086
GO:0006810	transport	0.3	0.001
GO:0051716	cellular response to stimulus	0.23	0.001
GO:0010647	positive regulation of cell communication	0.4	0.0011
GO:0007267	cell-cell signaling	0.54	0.0012
GO:0014910	regulation of smooth muscle cell migration	1.06	0.0012
GO:0023056	positive regulation of signaling	0.4	0.0012
GO:0050778	positive regulation of immune response	0.65	0.0012
GO:0071310	cellular response to organic substance	0.37	0.0014
GO:0048522	positive regulation of cellular process	0.23	0.0015
GO:0010033	response to organic substance	0.32	0.0016
GO:0032940	secretion by cell	0.67	0.0017
GO:0045087	innate immune response	0.6	0.0017
GO:0031347	regulation of defense response	0.59	0.0018
GO:0048523	negative regulation of cellular process	0.24	0.0018
GO:0044092	negative regulation of molecular function	0.46	0.002
GO:0002675	positive regulation of acute inflammatory response	1.35	0.0023
GO:0035336	long-chain fatty-acyl-CoA metabolic process	1.69	0.0028
GO:0006950	response to stress	0.29	0.0029
GO:0048661	positive regulation of smooth muscle cell proliferation	0.97	0.0029
GO:0042221	response to chemical	0.26	0.003
GO:0051179	localization	0.24	0.003
GO:0010872	regulation of cholesterol esterification	1.65	0.0033
GO:0023061	signal release	0.85	0.0034
GO:0002253	activation of immune response	0.77	0.0036
GO:0009615	response to virus	0.77	0.0036
GO:0070555	response to interleukin-1	0.93	0.004
GO:0001932	regulation of protein phosphorylation	0.4	0.0042
GO:0010817	regulation of hormone levels	0.57	0.0045
GO:0060259	regulation of feeding behavior	1.24	0.0045
GO:0080134	regulation of response to stress	0.42	0.0045
GO:0002376	immune system process	0.36	0.0047
GO:0050727	regulation of inflammatory response	0.69	0.0047

Table S3: Table of biological and cellular pathways with an FDR < 0.05 detected in STRING analysis from Fig. 7c.

#term ID	term description	strength	false discovery rate
GO:0051607	defense response to virus	1.48	0.00032
GO:0035458	cellular response to interferon-beta	1.98	0.00096
GO:0009617	response to bacterium	0.99	0.0026
GO:0035457	cellular response to interferon-alpha	2.19	0.0067
GO:0051707	response to other organism	0.79	0.0067
GO:0098542	defense response to other organism	0.88	0.0067
GO:0045087	innate immune response	0.93	0.0101
GO:0006955	immune response	0.78	0.0127
GO:0035634	response to stilbenoid	1.88	0.0127
GO:0009605	response to external stimulus	0.56	0.0278

Table S4: Table of biological and cellular pathways with an FDR < 0.05 detected in STRING analysis from Fig. 7d.

#term ID	term description	strength	false discovery rate
GO:0035456	response to interferon-beta	1.81	1.22E-06
GO:0009617	response to bacterium	0.93	8.63E-06
GO:0035458	cellular response to interferon-beta	1.84	8.63E-06
GO:0051704	multi-organism process	0.56	6.78E-05
GO:0051707	response to other organism	0.73	6.78E-05
GO:0009605	response to external stimulus	0.55	0.00017
GO:0009615	response to virus	1.1	0.0002
GO:0051607	defense response to virus	1.2	0.00032
GO:0045087	innate immune response	0.83	0.00078
GO:0006952	defense response	0.65	0.0012
GO:0006955	immune response	0.68	0.0013
GO:0034097	response to cytokine	0.7	0.0021
GO:0071345	cellular response to cytokine stimulus	0.73	0.0036
GO:0002252	immune effector process	0.85	0.0045
GO:0002376	immune system process	0.49	0.0157
GO:0035457	cellular response to interferon-alpha	1.82	0.0343
GO:0071310	cellular response to organic substance	0.45	0.0343
GO:0071305	cellular response to vitamin D	1.79	0.0358

Table S5: Cell culture and pharmacological reagents and their final concentrations

Cell Culture and Pharmacological Reagents		Final Concentration
IMDM	GIBCO	-
DMEM	Caisson Laboratories	-
FCS	Atlanta Biologicals	10% v/v
Penicillin/streptomycin	GIBCO	1% v/v
Dexamethasone	Sigma Aldrich	0.1uM
Insulin	Sigma Aldrich	5 µg/mL

Table S6: Antibodies used and their final concentrations for western blots.

Antibodies		Final Concentration
p-FAK Tyr397 (3283)	Cell Signaling	1/1000
FAK (sc-558)	Santa Cruz Biotechnology	1/500
LDHA (2012S)	Cell Signaling Technology	1/1000
Vinculin (E1E9V)	Cell Signaling Technology	1/1000
PARP (9542S)	Cell Signaling Technology	1/1000
Lamin A/C (sc-7292)	Santa Cruz Biotechnology	1/1000
Sun-1 (HPA008346)	Sigma Aldrich	1/1000
Sun-2 (ab87036)	Abcam	1/1000
Adiponectin (ADIPOQ) (PA1-054)	ThermoFischer Scientific	1/1000
β-Tubulin (D3U1W)	Cell Signaling Technology	1/1000

Table S7: Immunostaining antibodies and reagents and their final concentrations.

Immunostaining antibodies and Reagents		Final Concentration
Hoechst 33342	Thermo Scientific	1 µg/mL
Alexa Fluor 488 Phalloidin	Life Technologies	0.1µM
Lamin A/C (sc-7292)	Santa Cruz Biotechnology	1:300
Sun-1 (MABT892)	EMD Millipore	1:300
Sun-2 (IQ444)	Immuquest	1:300
